# Correlations between hormone levels and cardiovascular autonomic neuropathy in menopausal patients with diabetes mellitus

**DOI:** 10.12669/pjms.36.6.2088

**Published:** 2020

**Authors:** Guiling Zhang, Wei Wei, Bo Tan, Jingqin Liu

**Affiliations:** 1 Guiling Zhang, Department of Endocrinology, Baoding No.1 Hospital, Baoding, Hebei 071000, P.R. China; 2 Wei Wei, Department of Endocrinology, Baoding No.1 Hospital, Baoding, Hebei 071000, P.R. China; 3 Bo Tan, Department of Endocrinology, Baoding No.1 Hospital, Baoding, Hebei 071000, P.R. China; 4 Jingqin Liu, Department of Endocrinology, Baoding No.1 Hospital, Baoding, Hebei 071000, P.R. China

**Keywords:** Cardiac autonomic neuropathy, Hormone level, Menopause, Type-II diabetes mellitus

## Abstract

**Objective:**

To discuss the correlation between hormone levels and cardiovascular autonomic neuropathy (CAN) in menopausal patients with Type-2 diabetes mellitus (T2DM).

**Methods:**

This clinical research study was conducted at Department of Endocrinology, Baoding No. 1 Hospital from January 2016 to December 2017. In this study a total of 386 menopausal female patients with T2 DM were selected and classified into two groups according to the CAN function test: the CAN group (80 cases) and the DM group (306 cases). The Kupperman score (KI integral) was calculated for all participants in the study, and the following indexes were measured: body mass index (BMI), blood estrogen (E_2_), follicle-stimulating hormone (FSH), luteinizing hormone (LH), thyroid-stimulating hormone (TSH), free thyroxine (FT_4_), free triiodothyronine (FT_3_), fasting blood-glucose (FBG), glycosylated hemoglobin (HbA1c), serum lipids, uric acid (SUA), hypersensitive c-reactive protein (CRP), etc.

**Results:**

The FBG, HbA1c, TGs, Hs-CRP, SUA, KI score, TSH, FSH and LH of the CAN group were obviously higher than the same parameters in the DM group (P<0.01, P<0.05), while HDL-C, E_2_, FT_3_ and FT_4_ were significantly lower (Pπ.01, Pπ.05). Pearson correlation analysis indicated that CAN presents a positive correlation with HbA1c, TGs, hs-CRP and SUA and a negative correlation with HDL-C and E_2_, and the difference was statistically significant (P<0.05). The multifactor logistic regression analysis results showed that HbA1c (OR=3.980, 95%CI=1.268~10.319) and E2 (OR=3.075, 95%CI=1.167~7.366) are independent risk factors for CAN.

**Conclusion:**

The CAN morbidity of menopausal female patients with T2DM is high, and HbA_1_c and E_2_ should be mainly monitored to identify and treat CAN early.

## INTRODUCTION

Peripheral menopause syndrome (PMS) refers to a metabolic and neurological dysfunction syndrome in postmenopausal women characterized by reduced estrogen levels due to ovarian hypofunction, also called “menopausal syndrome”.^1^ Diabetic neuropathy is a common chronic complication of Type-I and Type-II diabetes. Cardiovascular autonomic neuropathy (CAN) is a kind of widely symmetrical polyneuropathy, which belongs to diabetic neuropathy and has important clinical significance. The autonomic nervous system includes sympathetic nervous system and parasympathetic nervous system. Their functions are independent and coordinated with each other. They can control heart rate, cardiac output, cardiovascular systolic and diastolic and electrophysiological activities.

Patients with T2DM may experience malignant arrhythmia and painless myocardial infarction and endangering life.^2^ There are only a few studies on the correlation between hormone level changes and CAN in menopausal patients with T2DM. In this study, the relationships among CAN, sex hormones and thyroid hormones in 386 female patients with T2DM were analyzed to explore the correlation between hormone levels and CAN.

## METHODS

### Ethical approval

The clinical research study was approved by the Institutional Ethics Committee of Baoding No. 1 Hospital at July 27^th^, 2019, and written informed consent was obtained from all participants.

### Case selection

A total of 386 45~55-year-old T2DM patients with PMS who were hospitalized at the Department of Endocrinology, Baoding No. 1 Hospital from January 2016 to December 2017 were included in this study, and the participants had an average age of 47.7±4.1 years. The duration of T2DM disease was 3-10 years, with an average duration of 5.9±2.5 years. The duration of amenorrhea was from three months to one year, with an average duration of (7.4±2.7) months . The CAN function test cut-offs were as follows: Valsalva test (VR) score ≤1.10, deep breathing heart rate difference (BD) ≤10 times/min, 30/15 specific value (LS) < 1.01, fist test (SHG) ≤10 mmHg, and postural hypotension (PH) >30 mmHg. The patients with 2 abnormal results in the above tests were classified into the CAN group (80 cases), and the others were classified into the DM group (306 cases). The age, BMI, duration of T2DM disease and duration of amenorrhea in both groups were not significantly different, and both groups were comparable.

### Inclusion criteria

All patients conformed to the 1999 DM diagnostic criteria of the WHO,^3^ received systematic treatment and complied with PMS diagnostic criteria.^4^ The duration of menstrual disorder exceeded three months or the occurrence of menopause was within one year and FSH was >10 IU/L. The improved Kupperman scale^5^ was ≥15, and the age ranged from 45-55 years old.

### Exclusion criteria:

Arrhythmia and myocardial ischemia changes visible on the routine electrocardiogram; use of β-receptor antagonist, atropine, digitalis and other drugs affecting heart rate within 2 weeks; acute complications of diabetes and severe chronic complications; acute infection; recent surgery, trauma or other stress states; bilateral ovariectomy or non-function; severe cardiac, cerebral, hepatic, renal or hemopoietic system diseases; severe hypertension; severe mental disease; sex hormone or thyroid hormone treatments within 3 months.

The 1999 T2DM diagnostic criteria of the WHO was used. Diabetic symptoms (typical symptoms include polydipsia, polyuria and unexplained weight loss) can be diagnosed by adding one of the following three items(1) Random blood glucose (refers to the blood glucose at any time of the day) ≥ 11.1 mmol / L (200mg%) (2) Fasting blood glucose (fasting state refers to no eating calories for at least 8 hours) ≥ 7.0 mmol / L (126 mg%) (3) 2 hours after glucose loading, blood glucose ≥ 11.1 mmol / L (200 mg%) without typical T2DM symptoms, reexamination is required.

### Diagnostic criteria for menopausal syndrome

Menopausal status was assessed clinically or based on serum hormone levels; that is, one year after the cessation of menstruation due to ovarian dysfunction is called menopause. Some scholars refer to it as perimenopause. The KI scoring scale was used to assess perimenopausal status.^5^ There are 13 symptoms, and the scoring is as follows: four points for hot flashes and perspiration; two points each for sensory disturbance, insomnia, easy excitation, urinary system infection and sexual life; and one point each for depression and suspicion, dizziness, fatigue, muscle arthralgia, headache, palpitation and formication. Degree of scoring: four levels were classified according to severity: 0 indicates no symptoms and scores of one, two and three indicate mild, moderate and severe symptoms, respectively. Symptom score = basic score of symptom x degree of scoring. The total score is the sum of the scores of each symptom. A KI score ≥15 indicates menopausal syndrome.

### Observation indicators

The subjects did not consume a high-fat diet and did not drink alcohol for three days before blood sampling. After fasting for 12 hour, 10 ml cubital venous blood was obtained in a vacuum blood collection tube without anti-freeze, and the time of tourniquet application on the arm was less than 30 s. A Hitachi 7600-110 fully automatic biochemical analyzer was used to test serum uric acid (SUA), FBG, total cholesterol (TC), triacylglycerol (TG), high-density lipoprotein (HDL-C), low-density lipoprotein (LDL-C), and high-sensitivity C-reactive protein (Hs-CRP). High-pressure liquid ion-exchange chromatography was used to measure glycosylated hemoglobin (HbA_1_C). Siemens ADVIA Centaur XP automatic chemiluminescence immunoanalyzer was used to measure sex hormones [E_2_, FSH and luteinizing hormone (LH)] and thyroid hormones [TSH, FT_4_ and FT_3_]. The Kupperman score (KI integral) was calculated, and the disease state was classified according to the score:^5^ mild: below 20 points, moderate: 21-35 points, and severe: above 35 points.

### Statistical analysis

SPSS 21.0 statistics software was used. The measurement data are expressed as ±s and were tested with a t-test. The enumeration data were tested with χ^2^. Pearson correlation analysis was adopted for correlation analysis. Multifactor logistic regression analysis was adopted for the influencing factors with statistical significance. The variable assignment is shown in Table-I. The test level was α=0.05.

**Table-I:** Variable assignment for logistic regression analysis of correlative factors between hormone level and CAN of T2DM patients in the menopause.

Influencing factor	Variable assignment
HbA_1c_	Measured value
TG	Measured value
hs-CRP	Measured value
SUA	Measured value
HDL-C	Measured value
E_2_	Measured value
Combined CAN	0=no, 1=yes

## RESULTS

The differences between both groups in age, BMI, amenorrhea duration and duration of diabetes were not statistically significant (P>0.05), as shown in Table-II.

**Table-II:** Comparison of general data (X̄±s)

Group	No.	Age (year)	BMI (kg/m2)	Amenorrhea time (m)	Course of disease (year)
CAN group	80	47.3±3.7	24.3±2.7	7.4±2.8	5.7±2.6
DM group	306	47.6±3.9	23.8±2.4	7.5±3.1	5.8±2.4
T value		0.6190	1.6155	0.2619	0.3260
P-value		0.5363	0.1070	0.7935	0.7446

FBG, HbA_1_c, TG, hs-CRP, SUA and the KI scores of the CAN group were higher than those of the DM group, and the HDL-C level was lower than that of the DM group. The differences were statistically significant (P<0.05). The differences between both groups in TC and LDL-C were not statistically significant (P>0.05), as shown in Table-III.

**Table-III:** Comparison of biochemical indicators and KI scores (X̄±s)

Group	No.	FBG(mmol/L)	HbA_1_c(%)	TC(mmol/L)	TG(mmol/L)	LDL-C(mmol/L)
CAN group	80	10.6±3.4	8.1±1.3	5.5±1.5	2.7±1.2	2.8±0.9
DM group	306	9.7±3.2	7.6±1.2	5.4±1.3	2.3±1.1	2.7±0.8
T-value		2.2107	3.2605	0.5927	2.8409	0.9693
P-value		0.0276	0.0012	0.5537	0.0047	0.3330

Group	No.	HDL-C(mmol/L)	hs-CRP(mg/L)		SUA(umol/L)	KI(score)

CAN group	80	0.8±0.2	1.5±0.4		268.6±51.5	28.8±9.7
DM group	306	0.9±0.3	1.4±0.3		254.9±48.2	25.6±8.4
T-value		2.8206	2.4047		2.2313	2.9348
P-value		0.0050	0.0142		0.0262	0.0035

E_2_, FT3 and FT4 of the CAN group were significantly lower than those of the DM group, and the difference was statistically significant (P<0.01, P<0.05). TSH, FSH and LH were significantly higher, and the difference was statistically significant (P<0.05), as shown in Table-IV.

**Table-IV:** Comparison of hormone level of hypophysis-gonad axis and hypophysis-thyroid axis (X̄±s)

Group	No.	E_2_(pmol/L)	LH(U/L)	FSH(U/L)	TSH(mIU/L)	FT_3_(pmol/L)	FT_4_(pmol/L)
CAN group	80	45.6±14.8	31.7±9.1	64.4±12.5	2.7421	4.4±1.0	15.7±3.2
DM group	306	50.8±15.5	29.3±8.7	60.7±11.6	2.0±0.6	4.6±1.2	16.6±3.4
T-value		2.6963	2.1653	2.4990	1.8±0.5	2.0566	2.1332
P-value		0.0073	0.0310	0.0129	0.0064	0.0404	0.0335

CAN was used as the dependent variable, and the duration of diabetes, HbA_1_c, FBG, TC, TGs, HDL-DL, LDL-C, SUA, Hs-CRP, LH, FSH, TSH, FT_3_ and FT_4_ were used as the independent variables for Pearson correlation analysis. The results showed that CAN presented a positive correlation with HbA1c, TGs, Hs-CRP and SUA and a negative correlation with HDL-C and E_2_, and the difference was statistically significant (P<0.05). CAN had no correlation with the duration of disease, FBG, TCH, LH, FSH, TSH, FT3 or FT4 (P>0.05), as shown in Table-V.

**Table-V:** Pearson correlation analysis of CAN and each indicator.

Item	HbA_1_c	TG	Hs-CRP	SUA	HDL-C	E_2_	LH	FSH
r	0.128	0.115	0.119	0.103	-0.134	-0.106	0.072	0.098
p	0.0118	0.0238	0.0194	0.0431	0.0084	0.0374	0.1580	0.0544

Multifactor logistic regression analysis was conducted for the factors with statistical significance, including HbA_1_c, TGs, hs-CRP, SUA, HDL-C and E_2_. The results showed that HbA_1_c and E_2_ were independent risk factors for CAN in menopausal women with diabetes, reaching statistical significance (P<0.05), as shown in Table-VI.

**Table-VI:** Multi-factor logistic regression analysis of hormone level and CAN of diabetic’s patients in the menopause.

Influencing factor	Β-value	SE	Wald value	P value	OR value (95%CI)
HbA1c	0.182	0.086	5.570	0.017	1.280 (0.268~8.319)
TG	0.578	0.091	11.933	1.338	0.869 (0.564~5.782)
hs-CRP	-4.920	1.258	89.535	0.463	0.815 (0.258~3.056)
SUA	-6.257	0.589	132.636	0.087	0.974 (0.902~1.095)
HDL-C	-7.353	0.456	0.312	0.571	0.695 (0.191~2.506)
E2	0.293	0.670	4.211	0.034	1.275 (0.167~7.366)

## DISCUSSION

CAN is a chronic complication of DM with high morbidity. At present, the pathogenesis is not completely clear. Some studies have indicated that CAN damage is related to neuron damage, glucose toxicity and reduction of neural blood flow^6^ and is caused by the interaction of multiple factors. For example, hyperuricemia is closely related to CAN. After intervention with benzbromarone, the SUA level of patients with T2DM and hyperuricemia was reduced, the inflammatory response was alleviated, and heart rate variability indicators improved.^7^ In this study, the FBG, HbA_1_c, TGs, hs-CRP and SUA of the CAN group were higher than those of the DM group, and the HDL-C level was lower than that of the DM group. These results indicate that except for glycometabolism disorder, SUA elevation, TG elevation and HDL-C reduction are also dangerous factors associated with CAN, which is consistent with the above studies. In addition, the KI score of the CAN group was obviously higher than that of the DM group, indicating that climacteric symptoms of diabetic menopausal patients combined with CAN are obviously exacerbated.

Estrogen is an important hormone participating in the metabolic regulation of the body. The ovarian function of climacteric patients declines, so the estrogen secretion level gradually decreases and even completely disappears.^8^ Due to the lack of estrogen, dyslipidemia, central obesity and a sharp increase in metabolic syndrome risk, the progression of T2DM and cardiovascular disease is accelerated.^9^ The metabolic disorders caused by T2DM make increase climacteric age of patients, and the probability of menopause occurrence before the age of 45 is 3-times higher than that of normal women, and the symptoms of menopause are aggravated.^10^ Many studies have shown that estrogen can enhance the activity of nitric oxide synthetase in nerve cells, improve NO concentration and lead to vasodilatation.^11^ Moreover, estradiol, as an antioxidant, can reduce the generation of mitochondria ROS and protect vascular endothelial cells through the mitochondrial pathway.^12^ In a mouse model of ovary castration, estrogen replacement treatment could inhibit ROS generation, increase respiratory function in the mitochondria and reduce nerve cell apoptosis.^13^ In this study, the E_2_ level of the CAN group was significantly lower than that of the DM group, and the FSH and LH levels increased. This result indicates that after menopause, the protective function of endogenous estrogen in the body is reduced, and the occurrence of CAN increases. Pearson correlation analysis showed that CAN was negatively correlated with the E2 level, indicating that estrogen is a protective factor against CAN. Multifactor logistic regression analysis results indicate that HbA_1_c and E_2_ are independent risk factors for CAN in menopausal women with diabetes. Hence, changes in serum HbA_1_c and E_2_ can be used as important indicators to evaluate the clinical symptoms and severity of menopausal women with diabetes.

Female patients with PMS lack estrogen, and their thyroid function is also influenced. In this study, the FT_3_ and FT_4_ of the CAN group were lower than those of the DM group, while the TSH level was higher than that of the DM group, indicating that the thyroid functions of the CAN group were lower than those of the DM group. The possible reasons are as follows: sex hormones and thyroid hormones are regulated together by the hypothalamus-hypophysis axis, and sex hormones and thyroid hormones are needed to maintain sexual function. As estrogen secretion is reduced, endocrine dyscrasia occurs. The functions of the hypophysis-thyroid axis are influenced, and thyroid functions change abnormally.

### Limitations of the study

However, the difference in the number of cases between CAN group and DM group is relatively large, and the error may be slightly larger. The next step is to narrow the difference between the two groups in the number of cases for further study.

## CONCLUSION

To allow diabetic patients to go through menopause in a better way, reduce Cardiac Autonomic Neuropathy (CAN) and relieve the trouble of menopause, it is required to screen thyroid hormone levels, eliminate the influence of thyroid function and better control climacteric symptoms, in addition to the examination of estrogen levels.

### Authors’ Contributions:

**GZ and JL** designed this study and prepared this manuscript.

**XX, WW and BT** collected and analyzed clinical data.

**FL and** **LH** significantly revised this manuscript.

**JL** is responsible and accountable for the accuracy or integrity of the work.
